# Securinine, a novel alkaloid, regulates cell cycle and EMT in gastric cancer by inducing iron-dependent cell death

**DOI:** 10.3389/fonc.2025.1599680

**Published:** 2025-09-10

**Authors:** Weiwei Yuan, Hao Song, Yin Shi, Jianye Han, Shaohe Jiao, Song Liang, Yuanmin Xu, Yi Jiang, Yuehui Ji, Zhangming Chen, Limin Liu, Aman Xu, Zhou Xu

**Affiliations:** ^1^ Department of Thyroid Surgery, Baoshan Hospital Affiliated to Shanghai University of Traditional Chinese Medicine, Shanghai, China; ^2^ Department of Internal Medicine, Yiwu Maternity and Children Hospital, Yiwu, China; ^3^ Department of General Surgery, First Affiliated Hospital of Anhui Medical University, Hefei, China; ^4^ Department of General Surgery, The Lu’an Affiliated Hospital of Anhui Medical University, Lu’an People’s Hospital, Lu’an, China; ^5^ Department of Anesthesiology, The First Affiliated Hospital of University of Science and Technology of China, Division of Life Sciences and Medicine, Hefei, China; ^6^ Nanjing University of Chinese Medicine, Nanjing, China; ^7^ Department of General Surgery, The First People’s Hospital of Wuhu, Wuhu, China

**Keywords:** securinine, gastric cancer, epithelial-mesenchymal transition, tumor cell cycle, iron metabolism pathways

## Abstract

**Background:**

Gastric cancer remains one of the most prevalent and lethal cancers worldwide, with its insidious onset hindering early diagnosis and effective treatment. Despite advances, the overall survival rate for gastric cancer remains low, primarily due to late diagnosis, tumor heterogeneity, and resistance to current therapies. This highlights the urgent need for novel therapeutic strategies.

**Methods:**

Gastric cancer cell lines were treated with securinine, followed by analysis of cell proliferation, cycle, and Epithelial-Mesenchymal Transition (EMT) using Western blot and immunofluorescence techniques. Transcriptomic analysis was performed to identify changes in ferroptosis-related iron metabolism pathways. *In vivo* studies were conducted using xenograft mouse models to assess tumor growth.

**Results:**

Securinine significantly inhibited proliferation and modulated the cell cycle, arresting cells at the G2/M transition, while also enhancing EMT, which altered cell migration and invasiveness. Transcriptomic analysis revealed that securinine activated ferroptosis-related iron metabolic pathways, upregulating key genes such as HMOX1, FTH1, and FTR. Inhibition of these genes reversed the effects on cell proliferation and EMT, highlighting the role of ferroptosis in the anticancer effects of securinine. *In vivo* studies demonstrated a significant reduction in tumor growth in xenograft models.

**Conclusions:**

Securinine shows potential as a novel therapeutic agent for gastric cancer by inducing ferroptosis and modulating key cell death and survival pathways. Its ability to regulate iron metabolism and EMT suggests that it could be a promising candidate for developing new therapeutic strategies against gastric cancer, especially for drug-resistant cases.

## Introduction

Gastric cancer remains one of the most prevalent and deadly malignancies worldwide, particularly affecting developing countries. In 2022, there were over 968,000 newly diagnosed cases of gastric cancer globally, with nearly 660,000 deaths, making it the fifth leading cancer in both incidence and mortality (1). Although gastric cancer is not the most common malignancy among men worldwide, it remains a major gastrointestinal cancer and imposes a substantial disease burden in certain high-incidence regions, particularly in East Asia (2). Despite advances in treatment, the overall five-year survival rate for gastric cancer remains low, primarily due to late diagnosis, significant tumor heterogeneity, and resistance to current treatment options (3–5). Traditional approaches, including surgery, chemotherapy, and radiotherapy, provide limited benefits in advanced cases (6–8), underscoring the urgent need for novel therapeutic strategies to improve patient prognosis.

Natural compounds have garnered considerable attention for their potential role in cancer treatment, with many compounds being investigated for their broad biological activity and anticancer properties (9–11). Securinine, a naturally occurring alkaloid derived from Securinega suffruticosa and other plant species, has historically been used primarily as a GABAA receptor antagonist for neurological disorders (12, 13). Recently, research has shifted to explore the anticancer potential of securinine, demonstrating significant efficacy in inhibiting the growth of various malignancies, including leukemia, breast cancer, and prostate cancer (14–17). The anticancer activity of securinine is mainly attributed to its ability to modulate several key signaling pathways, including PI3K/Akt/mTOR, Wnt, and JAK/STAT, which play crucial roles in cell proliferation, apoptosis, autophagy, and metastasis (18). Given its broad biological activity, securinine shows great promise as a targeted therapeutic agent for drug-resistant cancers. The current research aims to explore whether securinine exerts its anticancer effects on gastric cancer by modulating ferroptosis, a novel aspect being investigated in this study.

Ferroptosis is a unique form of regulated cell death characterized by iron-dependent lipid peroxidation, distinguishing it from traditional programmed cell death mechanisms such as apoptosis and necrosis. This specialized form of cell death involves iron accumulation and the subsequent production of reactive oxygen species (ROS), leading to widespread lipid peroxidation, ultimately causing membrane damage and cell death (19, 20). Inducing ferroptosis has been suggested as a potential strategy to overcome treatment resistance in cancer cells, making it an attractive target for treating various hard-to-treat malignancies (21, 22). Activation of ferroptosis through modulation of iron metabolism and lipid peroxidation pathways offers a novel, targeted strategy for treating gastric cancer, particularly in patients who exhibit resistance to conventional therapies. Natural compounds have shown potential in inducing ferroptosis (23, 24), thereby providing new avenues for gastric cancer treatment by effectively targeting iron metabolism and enhancing lipid peroxidation.

This study aims to explore the therapeutic potential of securinine in gastric cancer by investigating its role in inducing ferroptosis and regulating associated cellular processes such as epithelial-mesenchymal transition (EMT). By elucidating the molecular mechanisms underlying the anticancer effects of securinine, particularly its involvement in ferroptosis, this research seeks to identify novel therapeutic targets for gastric cancer. Ultimately, the findings of this study may contribute to the development of new, more effective treatment strategies that improve patient prognosis, reduce side effects, and enhance the quality of life for patients suffering from this aggressive disease.

## Methods

### Preparation and storage of securinine

Securinine (crystalline form, purity ≥ 98%, supplier: MCE [MedChemExpress]) was handled under strictly sterile conditions to prevent contamination. First, the crystals were accurately weighed with an analytical balance. Under a laminar-flow hood, the crystals were ground thoroughly with a sterilized ceramic mortar and pestle to obtain a homogeneous fine powder. The powder was immediately transferred to sterile, airtight amber-glass vials to protect the compound from moisture and direct light. The vials containing securinine powder were stored at −20°C to preserve chemical stability and bioactivity.

### Detailed integrated preparation procedure for securinine solutions for *in vitro* and *in vivo* experiments

Securinine crystals are first ground in a mortar to a fine powder, transferred to a sterile Eppendorf (EP) tube, sealed tightly with Parafilm, and stored at 4°C in the dark until use.

*In vitro* stock solution: Accurately weigh ~2.96 mg securinine (record the exact mass) and, based on this mass, calculate the required volume of dimethyl sulfoxide (DMSO). Using a micropipette, introduce the powder into 1 mL DMSO, then dissolve completely by repeated pipetting, vortex mixing, and brief ultrasonication. Aliquot the clear solution into 100 μL portions and store long-term at –80°C.

*In vivo* working solutions: Weigh the amount of securinine required by the dosing schedule on a high-precision analytical balance. Dissolve the drug in a PEG400: ethanol: 0.9% saline mixture (57.1: 14.3: 28.6, v/v) to obtain final concentrations of 2.5 mg/mL and 5 mg/mL. Vortex thoroughly, then sonicate for 30 min to ensure complete dissolution. Place the solution at –80°C overnight to promote thorough mixing; the next day, sonicate again to restore homogeneity. Divide the solution into seven equal portions for daily administration. On the day of dosing, warm the required aliquot to 37°C in a water bath; keep the remaining aliquots frozen at –80°C until use.

### Cell culture procedures

Human gastric cancer cell lines MGC-803 and HGC-27 were purchased from the Cell Bank of the Chinese Academy of Sciences (Shanghai, China). All cell lines were authenticated by short tandem repeat (STR) profiling conducted by Shanghai Yihe Applied Biotechnology Co., Ltd. Cells were cryopreserved after reaching appropriate confluence, following standard protocols. Briefly, cells at suitable confluence were detached using 500 μL of trypsin in a 6 cm dish, centrifuged at 800 rpm for 4 min, and resuspended in serum-free cryopreservation medium (20% DMSO, 60% FBS, 20% RPMI 1640; Gibco, USA). Cryovials were frozen stepwise: initially at 4°C (10–60 min), then −20°C (30–120 min), and finally at −80°C overnight before transfer to liquid nitrogen for long-term storage. Thawing was performed quickly in a 37°C water bath (4 min), followed by centrifugation at 800 rpm for 3 min to remove DMSO. Cells were then resuspended in FBS-containing medium, seeded into suitable culture dishes, and incubated at 37°C with 5% CO_2_. Subculturing was performed when cells reached approximately 80% confluence, involving PBS washing, trypsinization, centrifugation, resuspension, and incubation at 37°C in 5% CO_2_ with regular medium renewal.

The primary antibodies used in this study were as follows:

CDK2 and Cyclin D1 (CST, Cell Signaling Technology, USA); P21 (Abcam, UK); E-cadherin, N-cadherin, Vimentin, HMOX1, FTH1, FTL, and GPX4 (Proteintech, China).

### Cell transfection

Before plating, the cell growth and confluence were assessed to ensure the cells were healthy and ≥80% confluent. After trypsinization, the cells were centrifuged and gently resuspended to form a uniform single-cell suspension. For plasmid overexpression or siRNA silencing experiments, the cells were seeded into six-well plates to achieve 60%-80% confluence by the following day. After plating, the cells were evenly distributed using an “8”-shaped shaking motion and incubated. The next day, the cell density and growth were checked to confirm they met the transfection conditions. For transfection, Solution A was prepared by mixing 5 μL of Lipofectamine 3.0 with 245 μL of Opti-MEM and incubating for 5 minutes. Solution B was prepared by mixing 2 μg of plasmid DNA or 150 pmol of siRNA with 245 μL of Opti-MEM and incubating for 5 minutes. The siRNA sequences are provided in **Supplementary Table S1** of the **Supplementary Materials**. Solutions A and B were then combined and incubated for 20 minutes. Under sterile conditions in a laminar flow hood, the culture medium in the six-well plate was aspirated and replaced with PBS. Each well was then filled with serum-free medium. After the incubation period, 500 μL of the A-B mixture was added to each well. The plate was returned to the incubator and cultured for 4–6 hours. After this, the medium was replaced with complete medium without antibiotics, and the cells were cultured for an additional 24–48 hours. Protein or RNA was extracted for further analysis as required by the experiment.

### HE staining procedure

Tissue sections were first baked at 60°C for 20 minutes to fix the tissue, followed by deparaffinization in xylene for 20 minutes. The sections were then rehydrated stepwise in anhydrous ethanol, 95% ethanol, 90% ethanol, 80% ethanol, and 70% ethanol for 5 minutes each, and washed three times with distilled water. The sections were stained with hematoxylin for 3 minutes to stain the cell nuclei, rinsed three times with distilled water, and briefly immersed in 1% hydrochloric acid alcohol for 3 seconds, followed by three additional washes with distilled water. For cytoplasmic staining, the sections were then stained with eosin for 1 minute. After dehydration in anhydrous ethanol and xylene, the sections were air-dried and mounted for microscopic examination and photographic recording. This comprehensive procedure allowed for clear visualization of both the nuclear and cytoplasmic structures of the tissue sections.

### Immunohistochemical staining procedure

Tissue samples were fixed in 10% formalin for at least 24 hours to ensure proper fixation, followed by washing with distilled water for 2 hours to remove excess fixative. The samples were then dehydrated progressively using an automatic dehydrator, with ethanol concentrations increasing from low to high. After dehydration, the tissues were embedded in molten paraffin and allowed to cool and solidify in molds to form wax blocks. Using a microtome, the blocks were sectioned into 5-μm thick slices, which were then placed onto preheated (40°C) water to flatten. The sections were picked up with pre-warmed slides and transferred through xylene for wax removal (15 minutes each time). The slides were rehydrated in graded ethanol solutions (100%, 95%, 80%) for 5 minutes per step and then rinsed with deionized water. Antigen retrieval was performed by microwaving the sections in boiling citrate buffer for 2 minutes, followed by natural cooling to restore antigen structure altered during fixation. After cooling, the slides were washed with PBS to remove residual citrate buffer. To block endogenous peroxidase activity, the sections were treated with 3% H_2_O_2_ for 20 minutes. Non-specific binding sites were blocked by incubating the slides with 3% BSA for 60 minutes. The primary antibody (e.g., anti-ASAP2, diluted 1:500) was added and incubated at 37°C for 60 minutes. After washing with PBS, the secondary antibody, typically conjugated with an enzyme or fluorescent marker, was added and incubated for 30 minutes. The sections were developed with DAB and H_2_O_2_ substrate at room temperature for 1 minute to visualize the antigen-antibody binding sites. The sections were then counterstained with hematoxylin to stain the nuclei. After counterstaining, the slides were dehydrated through a graded alcohol series, cleared in xylene, and mounted with neutral resin. The sections were air-dried before observation. Finally, the stained slides were observed under a Zeiss optical microscope (Carl Zeiss, Oberkochen, Germany) to evaluate staining results. Immunostaining scores were determined by evaluating staining intensity and the percentage of positive areas. The scoring was performed by at least two experienced pathologists to ensure accuracy and reliability.

### Immunofluorescence experiment

On Day 1 and 2, cells were plated at a density of 2 × 10^5^ cells/well onto coverslips in 24-well plates to ensure uniform growth. The following day, cell attachment was checked under a microscope to ensure the cells were well spread, which is crucial for obtaining high-quality images. On Day 3, mitochondrial staining was performed using Mito-tracker. A 200 nM working solution was prepared according to the manufacturer’s instructions and pre-warmed to 37°C. 1 mL of the working solution was added to each well and incubated for 30 minutes for mitochondrial staining. The cells were then washed three times with PBS to remove excess Mito-tracker and fixed in -20°C methanol for 20 minutes (150 μL per well). After washing three times with PBS, the cells were permeabilized with PBS containing 0.5% Triton X-100 at room temperature for 20 minutes (150 μL per well). Following PBS washes, the cells were blocked with 10% BSA for 20 minutes (150 μL per well). After removing the blocking solution, the primary antibodies (ASAP2 and ARHGAP21, diluted 1:50) were added to the cells and incubated overnight at 4°C. On Day 4, secondary antibody incubation and nuclear staining were performed. The cells were washed three times with PBS on a shaker, then incubated with FITC-conjugated goat anti-mouse antibody (1:500 dilution) and Alexa Fluor 594-conjugated goat anti-rabbit antibody (1:100 dilution) at room temperature for 1.5 hours in the dark. If nuclear staining was required, DAPI was added for 10 minutes of incubation in the dark, followed by three PBS washes. Excess liquid around the edges of the coverslips was gently absorbed with absorbent paper, and 2 μL of anti-fade reagent was applied to the slides. The coverslip was placed onto the slide with the cell-facing surface down, and the edges were sealed with nail polish. Imaging was performed within 1 hour using a confocal microscope, or the slides were stored in the dark at 4°C for later analysis.

### Subcutaneous tumor xenograft model in mice

A total of 40 five-week-old Balb/c nude mice were randomly assigned to different experimental groups for statistical comparison. The HGC27 cell line was cultured to the logarithmic growth phase. The fresh complete medium was warmed to room temperature, and the old medium was removed from the culture dish. The cells were washed with 2 mL of PBS, followed by the addition of 1 mL of trypsin for 1 minute to detach the cells. After gently dissociating the cells from the culture dish, 2 mL of complete medium was added to stop the digestion process. The cells were then collected, washed with 3 mL of PBS, and centrifuged (400 g, 5 minutes). The supernatant was discarded, and the cells were resuspended in PBS. After cell counting, the cell concentration was adjusted to 5 × 10^6^ cells per mouse, with a total volume of 60 µL for injection.

Mice were anesthetized with a mixture of oxygen and 2% isoflurane for 2–5 minutes and then fixed on a heating pad using a modified latex glove as an anesthesia mask. Once fully anesthetized, 60 µL of the cell suspension was injected subcutaneously near the fourth mammary gland. After injection, the site was gently compressed with a sterile cotton swab to prevent leakage. Starting from day 3, mice were intraperitoneally injected with 25 mg/kg or 50 mg/kg of the drug, while the control group received an equal volume of solvent. The injections were administered daily for 22 days. Mouse body weight and tumor volume were recorded every two days.

### Animal euthanasia and tissue collection

At the end of the treatment period, mice were euthanized by cervical dislocation, and tumors were excised and photographed. Major organs, including the heart, liver, spleen, and kidneys, were also collected and fixed in 10% formalin along with the tumor tissues. Fixed tissues were processed using an automatic tissue dehydrator, embedding machine, and semi-thin paraffin sectioning. Hematoxylin and eosin (HE) staining and immunohistochemistry (IHC) were performed, with particular focus on Ki67 staining to assess tumor proliferation. Tumor growth curves were plotted based on tumor volume changes, and differences in tumor growth between treatment groups were compared. The therapeutic effects and potential mechanisms of the drug were evaluated through IHC and HE staining results.

### Molecular docking of securinine and HMOX1

Molecular Structure of securinine: The molecular structure of securinine was obtained from the PubChem database. Energy Minimization of securinine: The structure of securinine was optimized using the Molecular Operating Environment (MOE) software. The “Amber10:EHT” force field was applied for optimization to ensure the molecule was in its lowest energy stable state prior to docking.

Protein Crystal Structure Acquisition: The crystal structure of HMOX1 protein was retrieved from the Protein Data Bank (PDB). The protein structure file was then imported into MOE, where water molecules beyond 4.5 Å from the protein were removed, and protonation of the protein was performed. Energy minimization was conducted to ensure the protein structure was in an ideal stable state. Prediction of Ligand Binding Pocket: The Site Finder tool in MOE was used to predict potential ligand-binding pockets on the HMOX1 protein. This step aimed to identify the most likely regions of the protein for interaction with securinine.

Docking Setup: The pre-processed HMOX1 protein structure was set as the receptor, with the binding site defined by the Site Finder predictions. The small molecule ligand, securinine, was used after energy minimization.

### RNA sequencing

RNA sequencing was performed by Genedenovo Biotechnology Co., Ltd (Guangzhou, China) using libraries sequenced on the Illumina sequencing platform. Total RNA was extracted from HGC27 cells treated with DMSO and securinine at its IC50 concentration, with three biological replicates for each group.

### Data processing and analysis

Data were analyzed using GraphPad Prism software, and protein immunoblotting (Western Blot) and cell immunofluorescence imaging results were quantified using ImageJ software. To ensure the reproducibility and reliability of the results, data were obtained from at least three independent experiments.

The differences between two groups were evaluated using Student’s t-test. Results are presented as the mean ± standard error of the mean (SEM), which reflects the variability and stability of the data. The significance levels were defined as follows: *p < 0.05, **p < 0.01, ***p < 0.001, ****p < 0.0001. A p-value of less than 0.05 was considered statistically significant.

### Data availability

The datasets generated and analyzed during the current study are available from the corresponding author upon reasonable request. All the data supporting the findings of this study are included in the article and its **Supplementary Information Files**. Experimental data, including cell viability assays, Western blot analyses, immunofluorescence imaging, and transcriptomic data, are available for further review. Additionally, the raw sequencing data supporting the transcriptomic analysis can be accessed upon request from the authors.

## Results

### The chemical structure of securinine and its impact on gastric cancer cell activity

**Figure 1:**
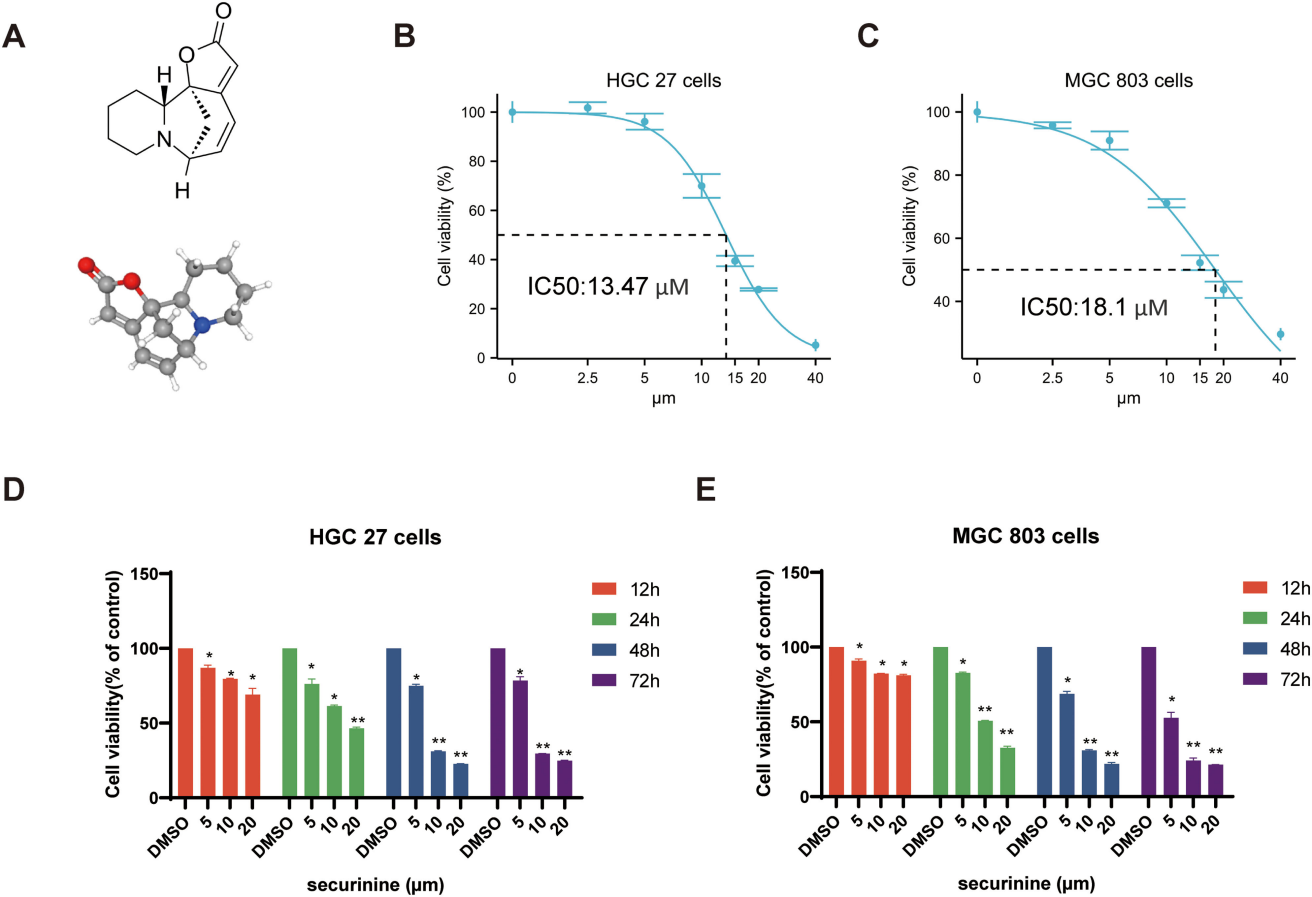
The molecular structure of securinine and its cytotoxic effects on two gastric cancer cell lines. **(A)**. Chemical molecular structure and 3D structure of securinine. **(B)**. HGC27 gastric cancer cells were treated with various concentrations of securinine for 24 hours, and cell viability was measured using the CCK-8 assay. **(C)**. MGC803 gastric cancer cells were treated with different concentrations of securinine for 24 hours, and cell viability was assessed using the CCK-8 assay. **(D)**. HGC27 gastric cancer cells were treated with different concentrations of securinine for 12, 24, 48, and 72 hours, and cell viability was determined using the CCK-8 assay. **(E)**. MGC803 gastric cancer cells were treated with varying concentrations of securinine for 12, 24, 48, and 72 hours, and cell viability was evaluated by the CCK-8 assay. The data represent the mean ± standard error of the mean (SEM) from three independent experiments. *P < 0.05, **P < 0.01, ***P < 0.001.

The chemical structure and 3D molecular structure of securinine are shown in **Figure 1A**. To investigate the cytotoxic effects of securinine on gastric cancer cells, human gastric cancer cell lines HGC27 and MGC803 were cultured and treated with various concentrations of securinine for different durations. The *in vitro* anticancer activity was assessed using the CCK-8 assay. The results revealed that the IC50 values of securinine for HGC27 and MGC803 cells were approximately 13.47 μM and 18.1 μM, respectively (**Figures 1B, C**). After 48 hours of treatment, securinine exhibited significant inhibitory effects on both gastric cancer cell lines at the IC50 concentration (**Figures 1D, E**).

### Morphological observation of HGC27 and MGC-803 gastric cancer cells treated with securinine

**Figure 2:**
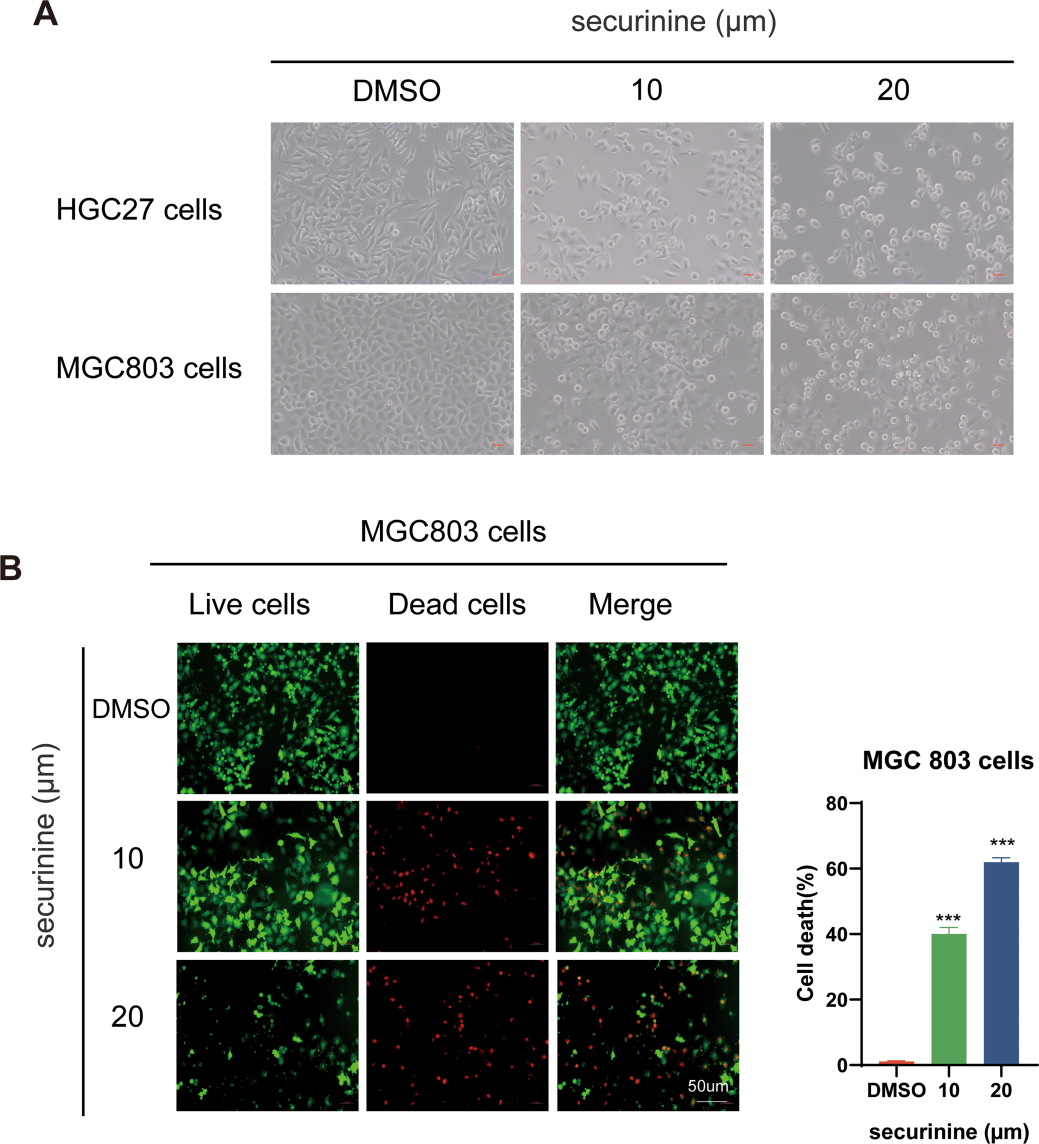
Morphological changes of HGC27 and MGC803 gastric cancer cells and live/dead staining of MGC803 Cells following securinine treatment. **(A)**. After 48 hours of treatment with securinine, the morphological changes and adhesion ability of HGC27 and MGC803 cells were observed under an inverted microscope. **(B)**. After 48 hours of securinine treatment, MGC803 cell viability and cytotoxicity were assessed using the Calcein/PI Live/Dead Cell Viability Assay Kit to detect changes in cell activity. The data represent the mean ± standard error of the mean (SEM) from three independent experiments. *P < 0.05, **P < 0.01, ***P < 0.001.

After 48 hours of treatment with securinine, the morphological characteristics of HGC27 and MGC-803 human gastric cancer cell lines were observed under an optical microscope. In the control group, cells exhibited healthy growth, characterized by a full shape, rapid proliferation, and tight attachment to the culture dish. In contrast, cells in the treatment group displayed clear signs of cell death, including shrinkage, rupture, and other morphological changes. Their proliferation rate and adhesion ability were significantly reduced. As shown in (**Figure 2A**), these observations highlight the significant inhibitory effect of securinine on gastric cancer cell growth.

Live/dead staining of MGC−803 cells confirmed this trend (**Figure 2B**). Following securinine treatment, the fraction of propidium−iodide–positive (dead) cells increased from ≈ 2 % in controls to ~40 % at 10 µM and ~65 % at 20 µM (n = 3), while viable, Calcein−AM–positive cells diminished accordingly. The strong green fluorescence of live cells and bright red fluorescence of dead cells clearly indicated intact versus compromised plasma−membrane integrity, respectively. Together, these results demonstrate that securinine induces substantial, dose−dependent cytotoxicity in gastric cancer cells.

### Securinine inhibits proliferation of gastric cancer cells

**Figure 3:**
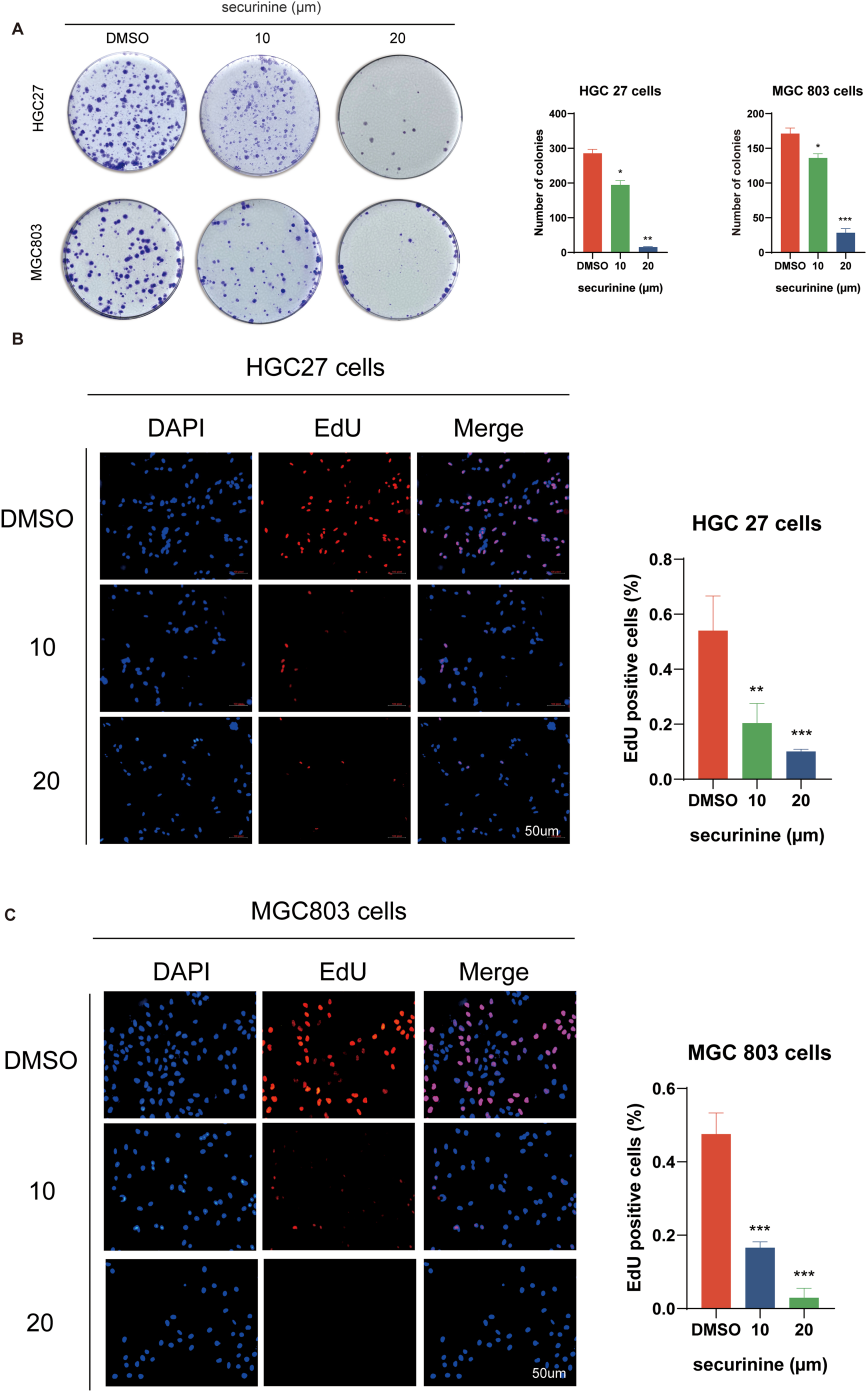
Inhibitory effect of securinine on clonogenic formation and EdU incorporation in gastric cancer cells HGC27 and MGC803. **(A)** Colony formation assays demonstrated a marked decrease in colony number upon securinine treatment at increasing concentrations. **(B, C)** EdU incorporation assays further confirmed reduced proliferation rates in both HGC27 and MGC803 cells treated with various concentrations of securinine. Data are presented as mean ± standard error of the mean (SEM) from three independent experiments. *P < 0.05, **P < 0.01, ***P < 0.001.

Colony−formation assays demonstrated that increasing concentrations of securinine markedly reduced clone numbers in both HGC−27 and MGC−803 gastric cancer cell lines (**Figure 3A**). In HGC−27 cells, 10 µM and 20 µM securinine suppressed mean colony numbers by approximately 57.5 % and 95.0 %, respectively (n = 3; 10 µM group P < 0.01, 20 µM group P < 0.001). A similar trend was observed in MGC−803 cells, with inhibition rates of about 58.3 % and 95.2 % (n = 3; both treatment groups P < 0.001).

EdU incorporation assays further verified the anti−proliferative effect of securinine (**Figures 3B, C**). In HGC−27 cells, treatment with 10 µM and 20 µM securinine reduced the percentage of EdU−positive nuclei by 62.0 % and 87.7 %, respectively (n = 3; P < 0.001). In MGC−803 cells, the decreases were 71.6 % and 92.9 %, respectively (n = 3; P < 0.001).

Taken together, securinine at 10–20 µM markedly inhibits colony formation and DNA synthesis in gastric cancer cells in a concentration−dependent manner, underscoring its potent anti−proliferative activity. All experiments were independently repeated three times, and data are presented as mean ± SD; all differences are statistically significant.

### Securinine induces G2/M phase cell-cycle arrest in HGC27 and MGC803 cells

**Figure 4:**
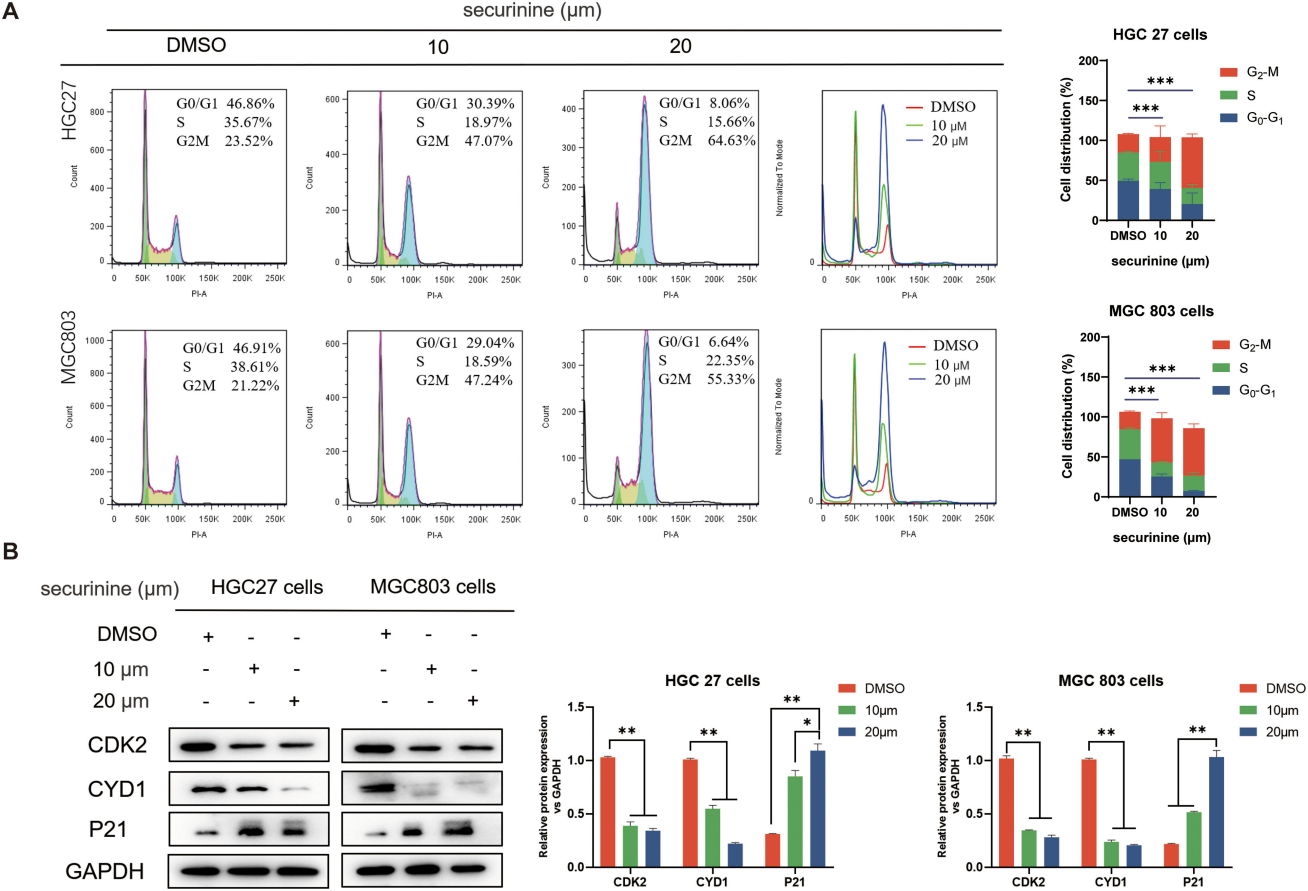
Securinine induces G2/M-phase arrest in HGC27 and MGC803 cells. **(A)**. HGC27 and MGC803 cells were treated with different concentrations of securinine, and flow cytometry was used to analyze the proportion of cells in the G1/G0, S and G2/M phases. The cell-cycle distribution is presented as bar charts. **(B)**. Western blot analysis of CDK2, Cyclin D1 and P21 expression in HGC27 and MGC803 cells treated with securinine. The data are presented as mean ± standard error of the mean (SEM) from three independent experiments. *P < 0.05, **P < 0.01, ***P < 0.001.

Compared with the control group, securinine treatment significantly reduced the proportion of HGC27 and MGC803 cells in the G1/G0 and S phases, while simultaneously increasing the population of cells arrested in the G2/M phase in a concentration-dependent manner (**Figure 4A**). These findings indicate that securinine blocks cell cycle progression at the G2/M checkpoint, causing cells to accumulate just before mitosis. Additionally, Western blot (WB) analysis of cell cycle-related proteins CDK2, Cyclin D1, and P21 (**Figure 4B**) revealed that the expression levels of CDK2 and Cyclin D1 were reduced, while P21 was markedly up-regulated in the securinine-treated groups relative to the control. Although CDK2 and Cyclin D1 are classically associated with G1-phase regulation, their down-regulation together with P21 up-regulation has also been reported in G2/M checkpoint activation. Taken together, these results suggest that securinine activates a damage-response program that halts cells at the G2/M boundary, thereby preventing the propagation of potentially compromised DNA.

### The effect of securinine on epithelial-mesenchymal transition in tumor cells

In this study, the effects of securinine on epithelial-mesenchymal transition (EMT) in tumor cells were explored. We employed cell immunofluorescence labeling and Western Blot (WB) techniques to further investigate the regulatory role of securinine on key EMT markers. EMT is a crucial mechanism for tumor cells to acquire invasive and migratory capabilities, characterized by the downregulation of epithelial markers and the upregulation of mesenchymal markers.

**Figure 5:**
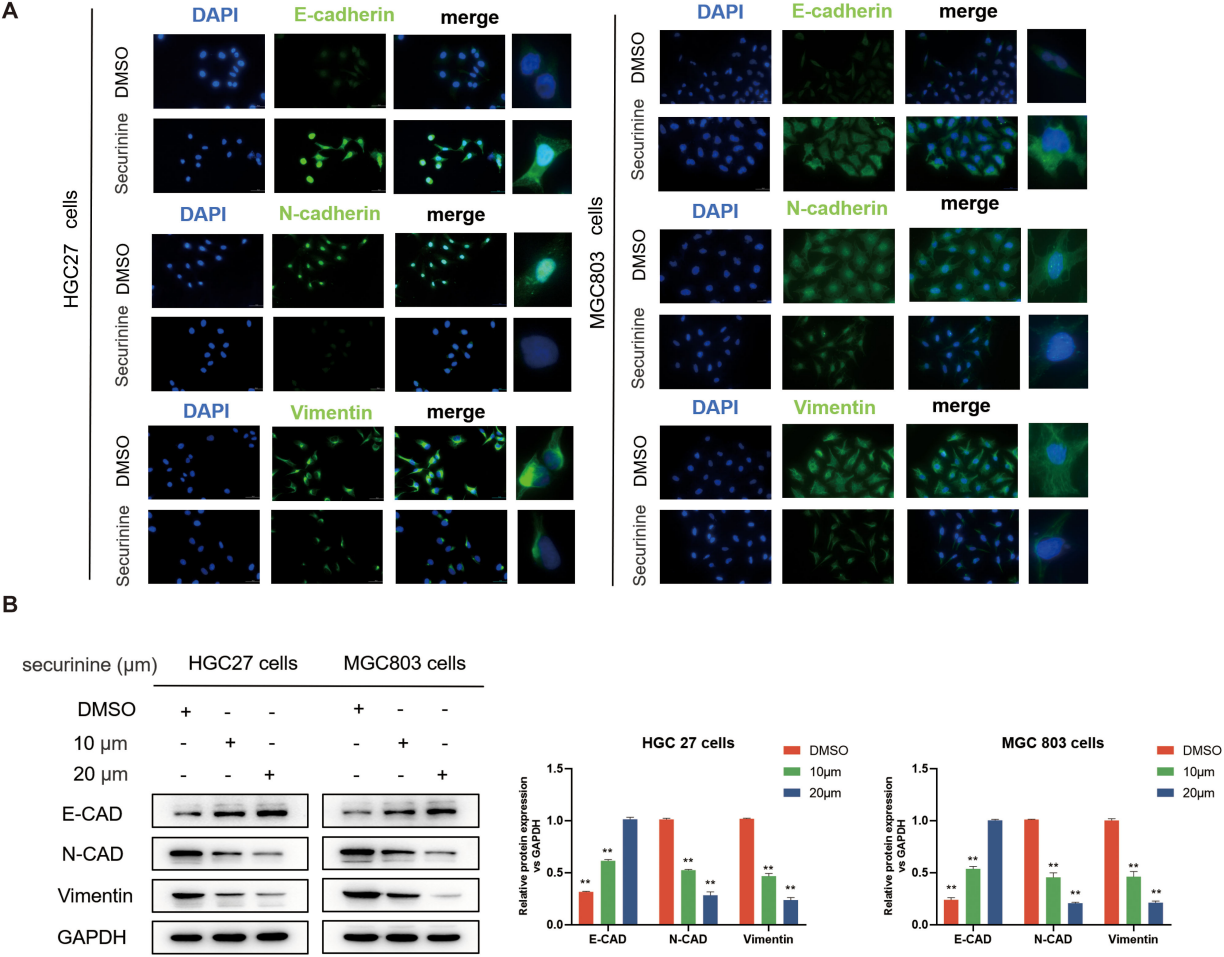
Securinine effectively inhibits the EMT process. **(A)** Immunofluorescence staining revealed that treatment with securinine at its IC_50_ concentration significantly increased the expression of the epithelial marker E-Cadherin (E-CAD) while notably decreasing the mesenchymal markers N-Cadherin (N-CAD) and Vimentin (VIM) in both HGC27 and MGC803 cells. **(B)** Western blot analysis further confirmed the concentration-dependent regulatory effects of securinine on these EMT-related proteins. Data represent the mean ± standard error of the mean (SEM) from three independent experiments. *P < 0.05, **P < 0.01, ***P < 0.001.

Cells were treated with securinine at its IC_50_ concentration (HGC27 cells 13.47 μM, 24h MGC803 cells 18.1 μM, 24h), after which immunofluorescence staining was performed to evaluate the epithelial marker E−Cadherin (E−CAD) and the mesenchymal markers N−Cadherin (N−CAD) and Vimentin (VIM) (**Figure 5A**), compared with the untreated control group, the securinine -treated group exhibited a significant increase in E-CAD expression, while the expression levels of N-CAD and VIM were notably decreased. These results directly indicate that securinine effectively inhibits the EMT process, blocking the transition of cells from an epithelial to a mesenchymal state and maintaining their epithelial characteristics.

To further validate these observations, WB analysis of the expression of E-CAD, N-CAD, and VIM was performed, as shown in (**Figure 5B**). Consistent results further strengthened the findings from the immunofluorescence experiments, confirming that securinine treatment significantly increased the expression of E-CAD, while simultaneously decreasing the levels of N-CAD and VIM, thus inhibiting the EMT process.

### *In vivo* efficacy evaluation of securinine in HGC27 gastric cancer nude mice

**Figure 6:**
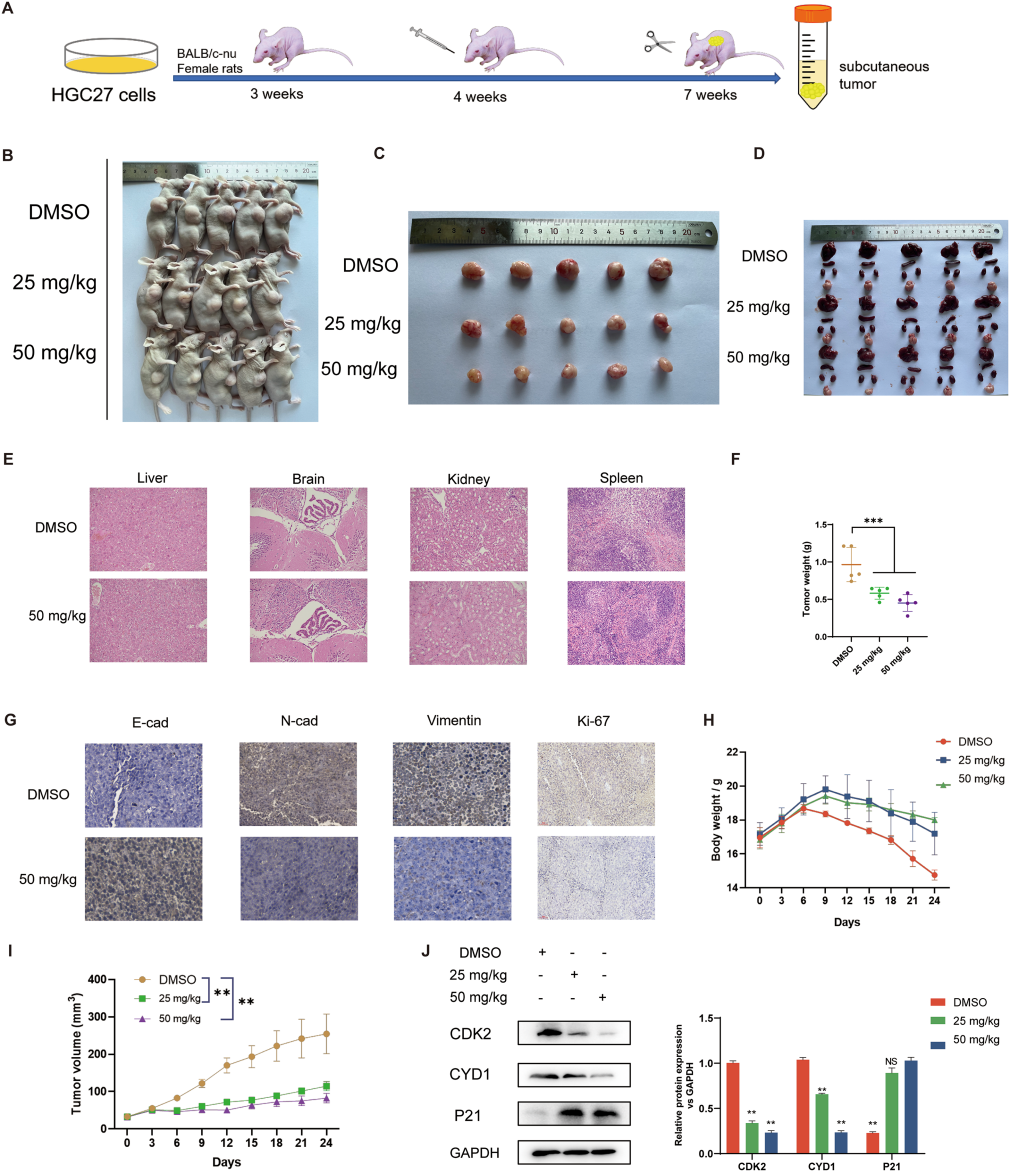
*In vivo* effects of securinine in mice. **(A)**. Schematic of the experimental protocol: 4-week-old female nude mice (5 per group) were subcutaneously injected with HGC-27 cells, followed by intraperitoneal injection of securinine at doses of 25 mg/kg and 50 mg/kg for 24 days. Mice were sacrificed at the end of the treatment. **(B)**. Representative images of nude mice with tumors under white light. **(C)**. Morphological changes in tumors after securinine treatment in tumor-bearing mice. **(D)**. Morphological analysis of the liver, spleen, kidneys, and heart in tumor-bearing mice after securinine treatment. **(E)**. HE staining of the liver, spleen, kidneys, and heart to assess the safety profile of securinine treatment in tumor-bearing mice. **(F)**. Differences in body weight of tumor-bearing mice after treatment. **(G)**. Immunohistochemical analysis of the expression of key proteins, including E-Cadherin (E-CAD), N-Cadherin (N-CAD), Vimentin (VIM), and Ki67 in HGC27 xenograft tumors. **(H)**. Dynamic body weight monitoring of tumor-bearing mice following securinine treatment. **(I)**. Dynamic tumor volume measurements in tumor-bearing mice after securinine treatment. **(J)**. Quantitative and qualitative analysis of cell cycle-related proteins CDK2, Cyclin D1, and P21 in HGC27 xenograft tumors using Western blot. Data are presented as mean ± standard error of the mean (SEM). NS indicates no significant difference, *P < 0.05, **P < 0.01, ***P < 0.001.

To evaluate the antitumor efficacy of securinine *in vivo*, we established a human HGC27 cell xenograft model using BALB/c nude mice (n=6 per group). Mice received daily intraperitoneal injections of DMSO (control), 25 mg/kg, or 50 mg/kg securinine for 24 consecutive days to assess the dose-dependent effect on tumor growth (**Figure 6A**). As shown in **Figures 6B, C**, securinine significantly suppressed tumor growth *in vivo*. Tumor volume measurements indicated that by day 24, average tumor volumes were reduced by approximately 60% (25 mg/kg group) and 72% (50 mg/kg group) compared to the control group (P<0.01; **Figure 6I**). Furthermore, tumor weights in the securinine-treated groups (25 mg/kg: ~0.6 g; 50 mg/kg: ~0.4 g) were significantly lower compared to the DMSO group (~1.1 g; P<0.001, **Figure 6F**).

For toxicity evaluation, mouse body weights were monitored throughout the treatment period, and HE staining was performed on major organs (liver, brain, kidney, and spleen) at the endpoint. There were no significant differences in body weight among groups (**Figure 6H**). Histopathological examination revealed no obvious signs of organ damage in any treatment group, indicating favorable tolerability (**Figures 6D, E**).

Additionally, immunohistochemical and Western blot analyses (n=3 per group) were conducted to elucidate the molecular mechanisms underlying securinine’s antitumor effects. Immunohistochemistry showed increased E-cadherin expression, decreased N-cadherin, Vimentin, and Ki67 staining intensity in tumors from securinine-treated mice (50 mg/kg group; **Figure 6G**), indicating suppression of epithelial-mesenchymal transition (EMT) and proliferation. Western blotting further validated these findings, showing securinine significantly reduced protein expression levels of proliferation markers CDK2 (~65% decrease) and CYD1 (~50% decrease), and upregulated cell cycle inhibitor P21 expression by approximately two-fold in treated groups compared to control (P<0.01, **Figure 6J**). These results collectively demonstrate that securinine effectively inhibits tumor growth *in vivo* via modulation of EMT and cell-cycle-related protein expression, with an acceptable safety profile.

### Transcriptomic profiling of gene expression changes in gastric cancer cells regulated by securinine

**Figure 7:**
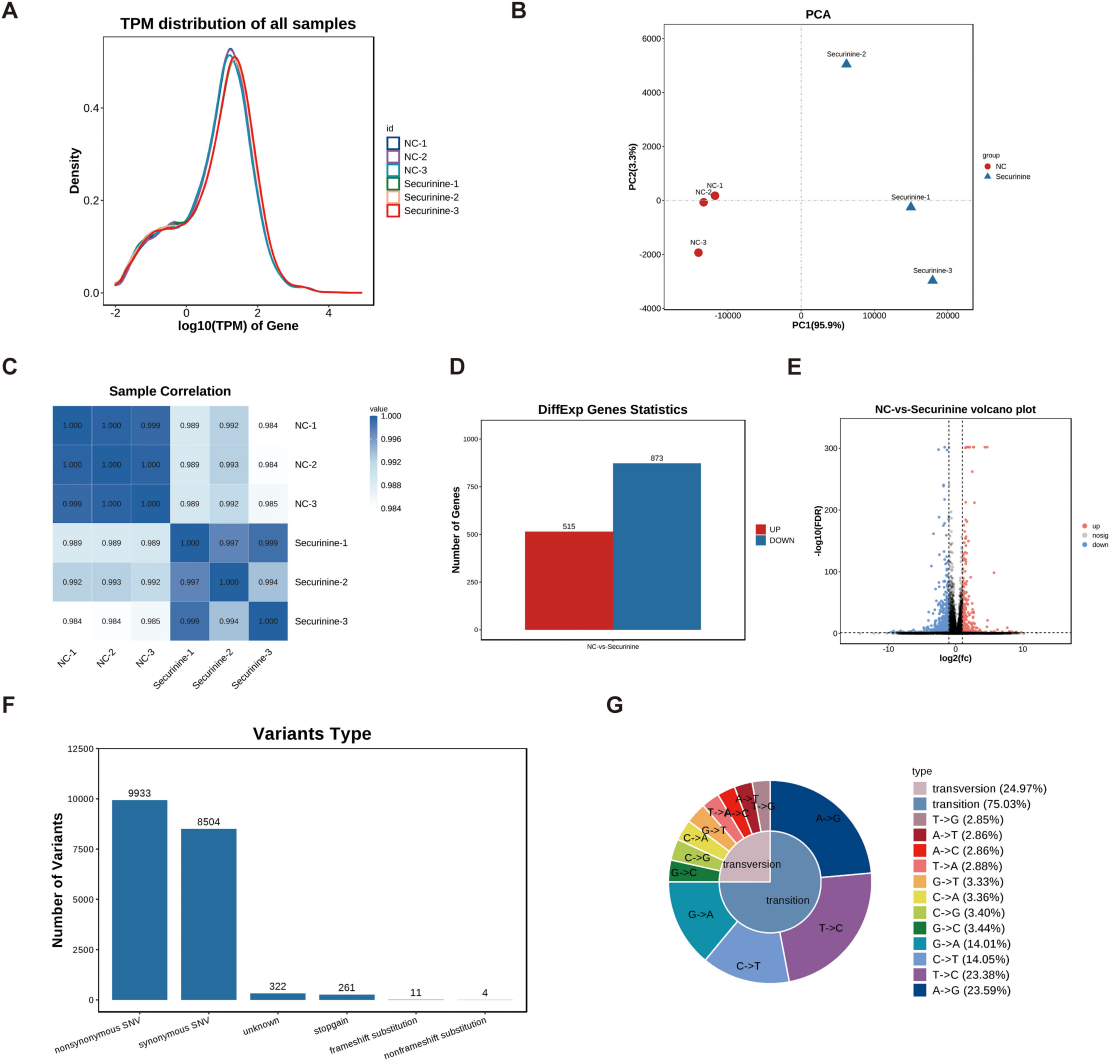
Transcriptomic profiling of gene expression changes in HGC27 gastric cancer cells treated with securinine. **(A)**. Gene expression distribution across different samples based on TPM values. The x-axis represents log10(tpm), with higher values indicating higher gene expression, while the y-axis represents gene abundance, calculated as the number of genes at a given expression level relative to the total number of detected genes. **(B)**. Principal component analysis (PCA) of gene expression in securinine-treated and control HGC27 cells. **(C)**. Correlation heatmap of gene expression between two randomly selected samples. Color intensity indicates the correlation coefficient, with blue representing higher correlation and white representing lower correlation. **(D)**. Heatmap of significantly differentially expressed genes between the control and securinine -treated groups (FDR < 0.05, |log2FC| > log2(2)). **(E)**. Volcano plot showing the distribution of differentially expressed genes. Genes located at the extremes of the plot exhibit more significant differences. **(F)**. SNP analysis of the sequencing data, focusing on nonsynonymous (nonsynonymous SNVs) and synonymous (synonymous SNVs) mutations. **(G)**. Statistical analysis of SNP mutation types, presented in a distribution chart.

To explore the regulatory effects of securinine on gene expression, we performed high-throughput transcriptomic screening of human HGC27 gastric cancer cells treated with DMSO and securinine at its IC50 concentration. After data quality control, we used the TPM (transcripts per million) values of each gene to display the expression distribution of genes or transcripts across different samples (**Figure 7A**). The x-axis represents log10(tpm), where higher values indicate higher gene expression levels, while the y-axis represents gene abundance, defined as the number of genes at a given expression level relative to the total number of detected expressed genes.

Based on these expression data, we conducted Principal Component Analysis (PCA) using R software (http://www.r-project.org/) (**Figure 7B**). To assess the correlation between different samples (Samples which tumor regression were induced by the innate immune response or instances of accidental mortality were excluded from the analysis), we performed a heatmap analysis of the gene expression levels from two randomly selected samples (**Figure 7C**), with color intensity indicating the correlation coefficient. Darker blue indicates higher correlation, while white indicates lower correlation.

Differential gene expression analysis was performed under the following selection criteria: FDR < 0.05 and |log2FC| > log2(2). A heatmap and volcano plot were generated for the significantly differentially expressed genes. The results showed that, compared to the control group, securinine treatment led to significant transcriptional changes in 1388 genes in HGC27 cells, with 515 genes upregulated and 873 genes downregulated (**Figure 7D**). Volcano plot analysis of these differentially expressed genes revealed that genes located at the extremes of the plot exhibited more significant differences (**Figure 7E**).

Finally, we performed SNP analysis on the sequencing data, focusing on non-synonymous mutations (nonsynonymous SNVs) and synonymous mutations (synonymous SNVs) (**Figure 7F**). We further categorized the SNP mutation types and presented the distribution of SNP mutations in a statistical chart (**Figure 7G**). These analyses provide a comprehensive view of the gene expression and mutation profiles altered by securinine treatment in gastric cancer cells.

### Transcriptomic screening reveals that securinine is primarily enriched in the ferroptosis and iron ion metabolism pathways

**Figure 8:**
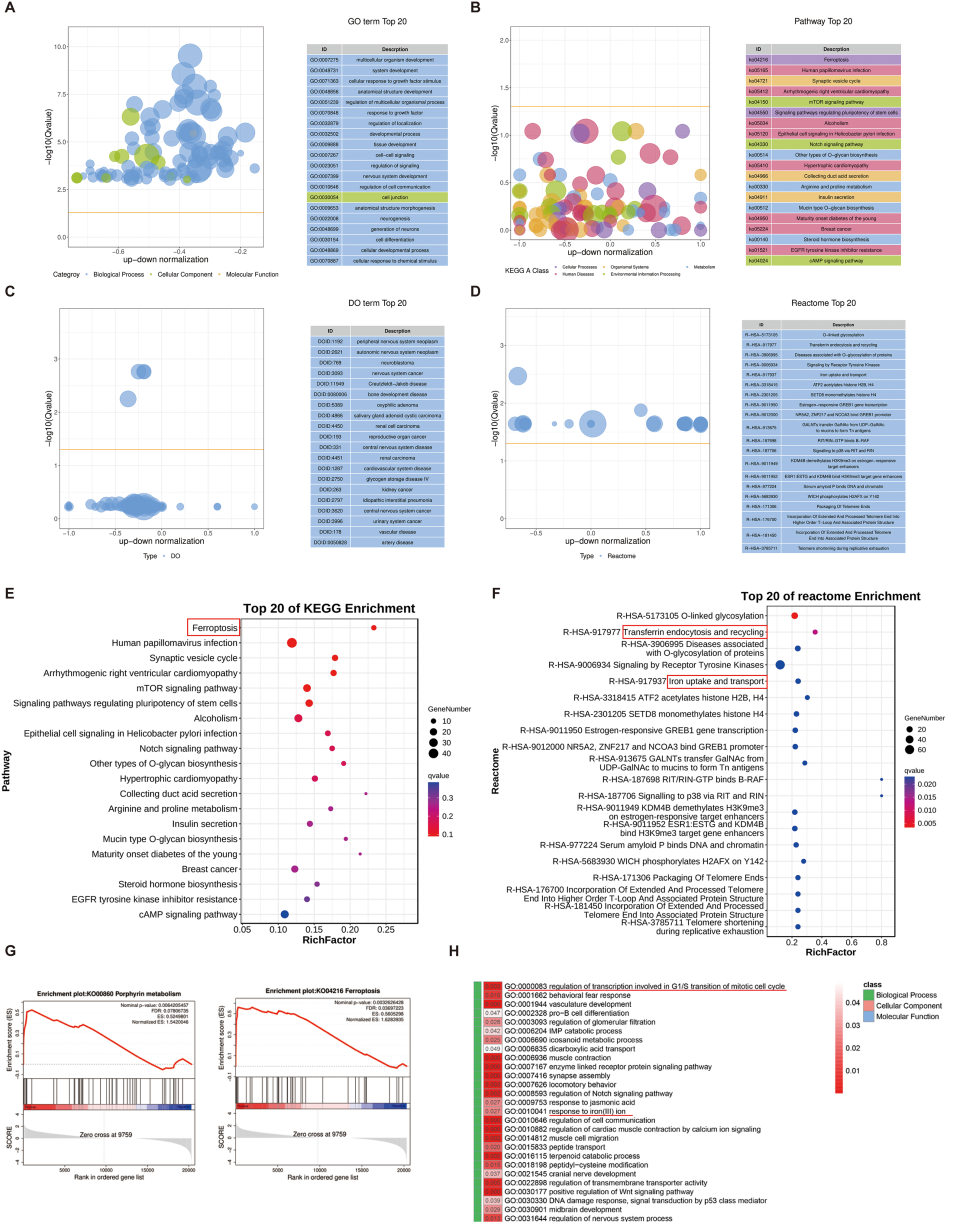
Enrichment analysis of differentially expressed genes in response to securinine. **(A)**. GO enrichment analysis bubble chart showing the top 20 enriched GO terms related to biological processes, cellular components, and molecular functions in securinine-treated HGC27 cells. **(B)**. KEGG enrichment analysis bubble chart displaying the top 20 enriched pathways based on Q-values. **(C)**. DO enrichment analysis bubble chart showing the top 20 enriched Disease Ontology (DO) terms. **(D)**. Reactome enrichment analysis bubble chart showing the top 20 enriched Reactome pathways based on Q-values. **(E)**. KEGG pathway analysis indicating that differentially expressed genes are primarily enriched in the ferroptosis pathway. **(F)**. Reactome analysis showing the enrichment of differentially expressed genes in transferrin endocytosis and iron uptake/transport pathways, essential for cellular iron homeostasis and function. **(G)**. GSEA analysis revealing that significantly differentially expressed genes promote iron metabolism and ferroptosis pathways. **(H)**. GO analysis of biological processes, cellular components and molecular functions showing that securinine affects cell-cycle regulation at the G2/M transition (mitotic checkpoint) and iron-ion-metabolism pathways.

To further investigate the biological processes regulated by securinine, we performed KEGG enrichment analysis, Reactome pathway analysis, and Gene Ontology (GO) enrichment analysis on differentially expressed genes. The top 20 GO terms are displayed in a differential GO enrichment bubble chart (**Figure 8A**), followed by KEGG enrichment analysis, which highlights the top 20 pathways based on Q-values (**Figure 8B**). Similarly, a differential Disease Ontology (DO) enrichment bubble chart shows the top 20 DO terms (**Figure 8C**), and a differential Reactome enrichment bubble chart presents the top 20 Reactome pathways (**Figure 8D**).

In KEGG enrichment analysis, we found that the differentially expressed genes were primarily enriched in the ferroptosis pathway (**Figure 8E**). Reactome pathway analysis further revealed that the differentially expressed genes were significantly enriched in transferrin endocytosis, as well as iron uptake and transport (**Figure 8F**). Iron uptake and transport are critical for oxygen transport, DNA synthesis, the electron transport chain, and the activity of numerous enzymes. Transferrin is internalized into cells through clathrin-mediated endocytosis, followed by recycling and reuse within the cell, a process essential for effective iron utilization and the regulation of intracellular iron ion concentrations.

Furthermore, Gene Set Enrichment Analysis (GSEA) indicated that the significantly differentially expressed genes promoted pathways related to heme biosynthesis and ferroptosis (**Figure 8G**). Finally, GO analysis of securinine’s biological effects in terms of biological processes, cellular components, and molecular functions revealed that securinine affects the regulation of transcription during specific phases of the cell cycle, particularly the G2/M transition, and modulates iron−ion−metabolism pathways (**Figure 8H**).

In summary, our findings suggest that securinine not only triggers cell‐cycle arrest at the G2/M transition, but also regulates iron ion pathways, promoting ferroptosis. These results lead us to hypothesize that securinine may affect gastric cancer cell function by inducing ferroptosis.

### Securinine promotes ferroptosis by influencing the key gene HMOX1 in the iron ion metabolism pathway

**Figure 9:**
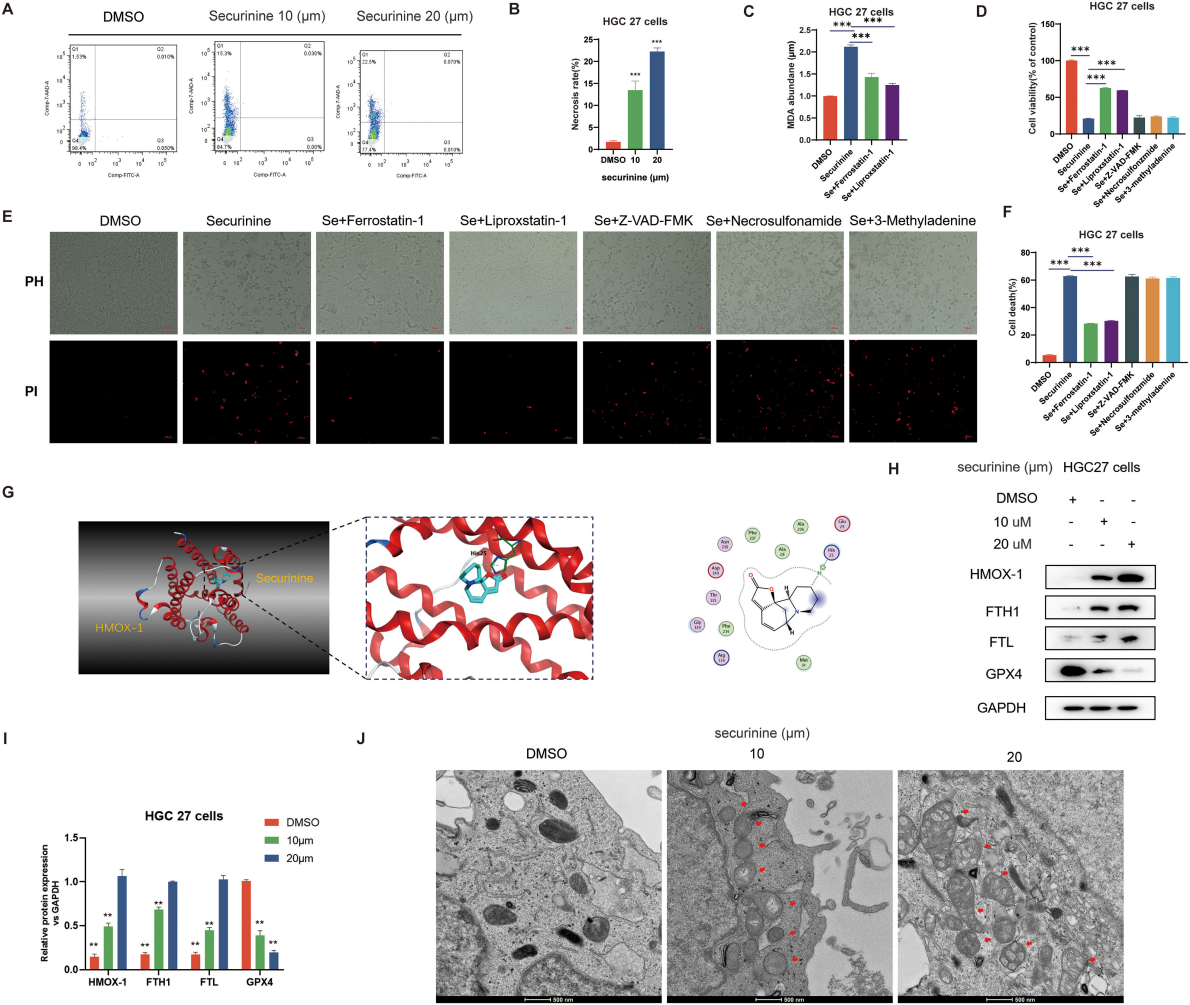
Securinine induces ferroptosis in gastric cancer cells by modulating HMOX1-mediated iron metabolism pathways. **(A)**. Flow cytometry analysis showing the effect of securinine on cell death, with a significant increase in necrosis. **(B)**. Bar chart representing the statistical data of cell death analysis from flow cytometry. **(C)**. Malondialdehyde (MDA) assay indicating that securinine treatment significantly increases intracellular iron accumulation, leading to elevated MDA levels, which decrease upon ferroptosis inhibitor treatment. **(D)**. CCK-8 assay evaluating the effect of ferroptosis inhibitors (Ferrostatin-1, Liproxstatin-1), apoptosis inhibitor (Z-VAD-FMK), necrosis inhibitor (Necrosulfonamide), and autophagy inhibitor (3-MA) on cell viability in securinine-treated HGC27 cells. **(E)**. Live/dead staining assay assessing the viability of securinine-treated HGC27 cells, showing cell death. **(F)**. Bar chart quantifying cell death data from the live/dead staining assay. **(G)**. Molecular docking analysis of the interaction between securinine and HMOX-1. **(H)**. Protein expression levels of HMOX-1, FTH1, FTL, and antioxidant protein GPX4 in HGC27 cells treated with securinine. **(I)**. Bar chart presenting the protein expression levels of HMOX-1, FTH1, FTL, and GPX4. **(J)**. Transmission electron microscopy images showing mitochondrial morphological changes in securinine-treated HGC27 cells, including swelling, reduced cristae, and increased mitochondrial membrane permeability, in a concentration-dependent manner. Data are presented as mean ± standard error of the mean (SEM). *P < 0.05, **P < 0.01, ***P < 0.001.

To elucidate the specific mechanism by which securinine induces cell death in HGC-27 cells, we first conducted flow cytometry analysis. Results demonstrated that securinine did not significantly trigger classical apoptosis, but rather increased the necrosis rate from approximately 5% (control) to ~14% at 10 µM and ~23% at 20 µM (an approximately 3–5-fold increase, n = 3; **Figures 9A, B**). Subsequently, malondialdehyde (MDA) assays indicated that 10 µM and 20 µM securinine elevated MDA levels by approximately 1.8-fold and 2.2-fold compared to the DMSO control. Treatment with ferroptosis inhibitors Ferrostatin-1 or Liproxstatin-1 reduced MDA levels by about 35%, whereas apoptosis inhibitor Z-VAD-FMK, necrosis inhibitor Necrosulfonamide, and autophagy inhibitor 3-MA showed minimal effects (**Figure 9C**). The CCK-8 assay further revealed that securinine treatment alone reduced cell viability to ~30% of control, whereas combined treatment with Ferrostatin-1 or Liproxstatin-1 partially restored viability to ~60% (**Figure 9D**). PI/PH live/dead staining consistently demonstrated that only ferroptosis inhibitors significantly decreased the proportion of PI-positive cells from about 70% to ~35% (**Figures 9E, F**). All these *in vitro* experiments were independently repeated three times.

Transcriptomic analysis suggested significant upregulation of key iron metabolism genes (**Figure 9I**). RT-qPCR results confirmed that mRNA levels of HMOX-1, FTH1, and FTL were elevated approximately 8–9-fold, 7–8-fold, and 6–7-fold, respectively, following 20 µM securinine treatment, whereas expression of the antioxidant enzyme GPX4 was reduced to about 15% of the control level (n = 3). These findings were corroborated by Western blotting analysis (**Figure 9H**). Molecular docking studies demonstrated stable binding of securinine to the active pocket of HMOX-1 (**Figure 9G**), suggesting potential direct regulation of heme degradation. Transmission electron microscopy further revealed securinine-induced mitochondrial swelling, reduced cristae, and decreased membrane density. The number of damaged mitochondria increased approximately fourfold relative to the control, exhibiting a concentration-dependent manner (**Figure 9J**).

In summary, securinine promotes intracellular iron accumulation by upregulating key iron metabolism-related proteins such as HMOX-1, subsequently triggering lipid peroxidation and depletion of GPX4. Thus, securinine induces HGC-27 cell death through ferroptosis rather than apoptosis.

### HMOX1 silencing and zinc protoporphyrin inhibitor application in iron-dependent cell death, gastric cancer cell cycle regulation, and the restoration of key protein expression in epithelial-mesenchymal transition

**Figure 10:**
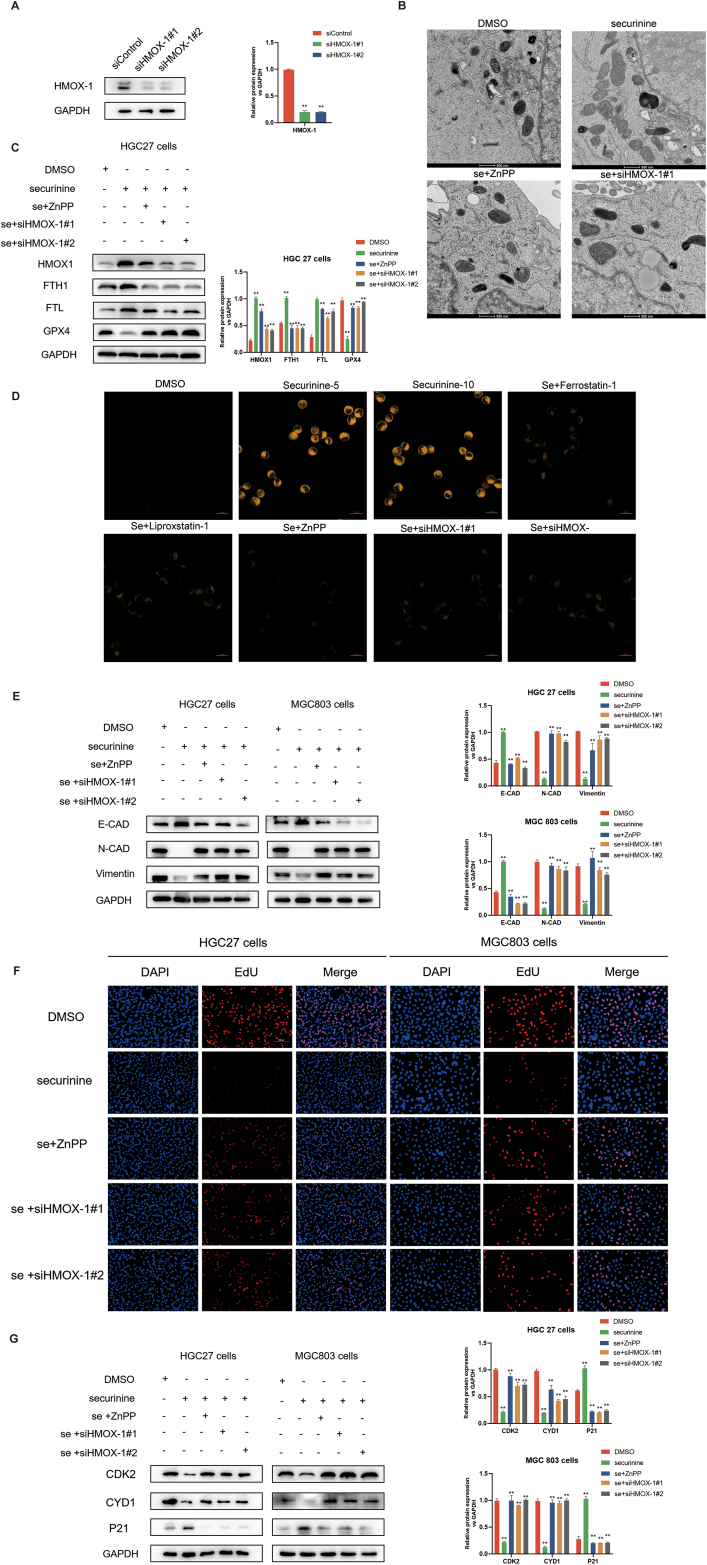
Effect of HMOX1 silencing and zinc protoporphyrin (ZNPP) inhibitor on iron-dependent cell death, gastric cancer cell cycle regulation, and EMT process in HGC-27 cells. **(A)**.Verification of HMOX1 gene silencing efficiency in HGC-27 cells using Western Blot, with the two most effective siRNA sequences selected for further experiments. **(B)**.Electron microscopy showing the effect of securinine treatment on mitochondrial morphology in HGC-27 cells. Mitochondrial swelling, reduced cristae, and increased membrane permeability were observed, with partial recovery upon application of ZNPP or HMOX1 gene silencing. **(C)**.Expression of iron death-related proteins, including HMOX1, FTH, FTL, and GPX4, following securinine treatment, ZNPP application, and HMOX1 silencing, assessed by Western Blot. **(D)**.Fluorescence imaging of lipid peroxides in live HGC-27 cells treated with securinine, ZNPP, or HMOX1 siRNA, using the lipid peroxide probe Liperfluo. **(E)**.Expression of EMT-related proteins (E-CAD, N-CAD, and VIM) in HGC-27 cells treated with securinine, ZNPP, or HMOX1 siRNA, analyzed by Western Blot. **(F)**. EdU assay to assess the proliferation of HGC-27 and MGC803 cells following securinine treatment, ZNPP application, or HMOX1 silencing. **(G)**. Western Blot analysis of cell cycle-related proteins (CDK2, Cyclin D1, P21) in HGC-27 cells treated with securinine, ZNPP, or HMOX1 siRNA. Data are represented as mean ± standard error of the mean (SEM). *P < 0.05, **P < 0.01, ***P < 0.001.

In this study, we investigated the role of HMOX1 gene silencing and the application of zinc protoporphyrin (ZnPP), a known HMOX1 inhibitor, in regulating ferroptosis, cell cycle progression, and epithelial-to-mesenchymal transition (EMT) in gastric cancer cells. Initially, we transiently transfected HGC-27 cells with two distinct siRNAs targeting HMOX1, and Western blot analysis confirmed effective knockdown, reducing HMOX1 protein levels by approximately 75–85% (**Figure 10A**).

Electron microscopy observations demonstrated mitochondrial swelling, reduced cristae, and increased mitochondrial membrane permeability upon securinine treatment, indicative of ferroptosis. Notably, co-treatment with ZnPP or silencing of HMOX1 markedly alleviated mitochondrial damage, partially restoring normal mitochondrial morphology (**Figure 10B**).

To further clarify the underlying mechanism, we assessed the expression levels of ferroptosis-related proteins. Securinine treatment significantly elevated HMOX1 protein expression by ~3-fold, while simultaneously downregulating FTH1 (~50% reduction), FTL (~60% reduction), and GPX4 (~65% reduction). These protein expression changes were notably reversed by ZnPP application or HMOX1 knockdown, indicating that securinine-induced ferroptosis relies heavily on the HMOX1 pathway (**Figure 10C**). Additionally, lipid peroxidation fluorescence staining using Liperfluo showed significantly increased lipid peroxide levels upon securinine treatment, whereas co-treatment with ZnPP or HMOX1 silencing markedly decreased lipid peroxidation signals (**Figure 10D**).

We further explored EMT-related protein changes. Western blot analysis revealed that securinine significantly increased epithelial marker E-cadherin expression (~2-fold increase), while decreasing mesenchymal markers N-cadherin (~70% reduction) and Vimentin (~60% reduction). These effects were substantially reversed following ZnPP treatment or HMOX1 gene silencing (**Figure 10E**).

Cell proliferation assays (EdU staining) demonstrated that securinine substantially suppressed proliferation in both HGC-27 and MGC803 cells, reducing EdU-positive cell proportions by approximately 75–85%. However, this suppression was significantly attenuated when combined with ZnPP treatment or HMOX1 knockdown (**Figure 10F**). Furthermore, Western blot analysis of cell cycle proteins revealed that securinine markedly decreased CDK2 and Cyclin D1 expression by ~50–65% and enhanced P21 expression approximately 2.5-fold. These alterations were also significantly reversed following ZnPP treatment or HMOX1 silencing (**Figure 10G**).

Collectively, our findings demonstrate that securinine induces ferroptosis, suppresses EMT, and inhibits gastric cancer cell proliferation predominantly through activation of the HMOX1-dependent ferroptosis pathway. These data highlight the critical regulatory role of HMOX1 in determining gastric cancer cell fate.

**Figure 11:**
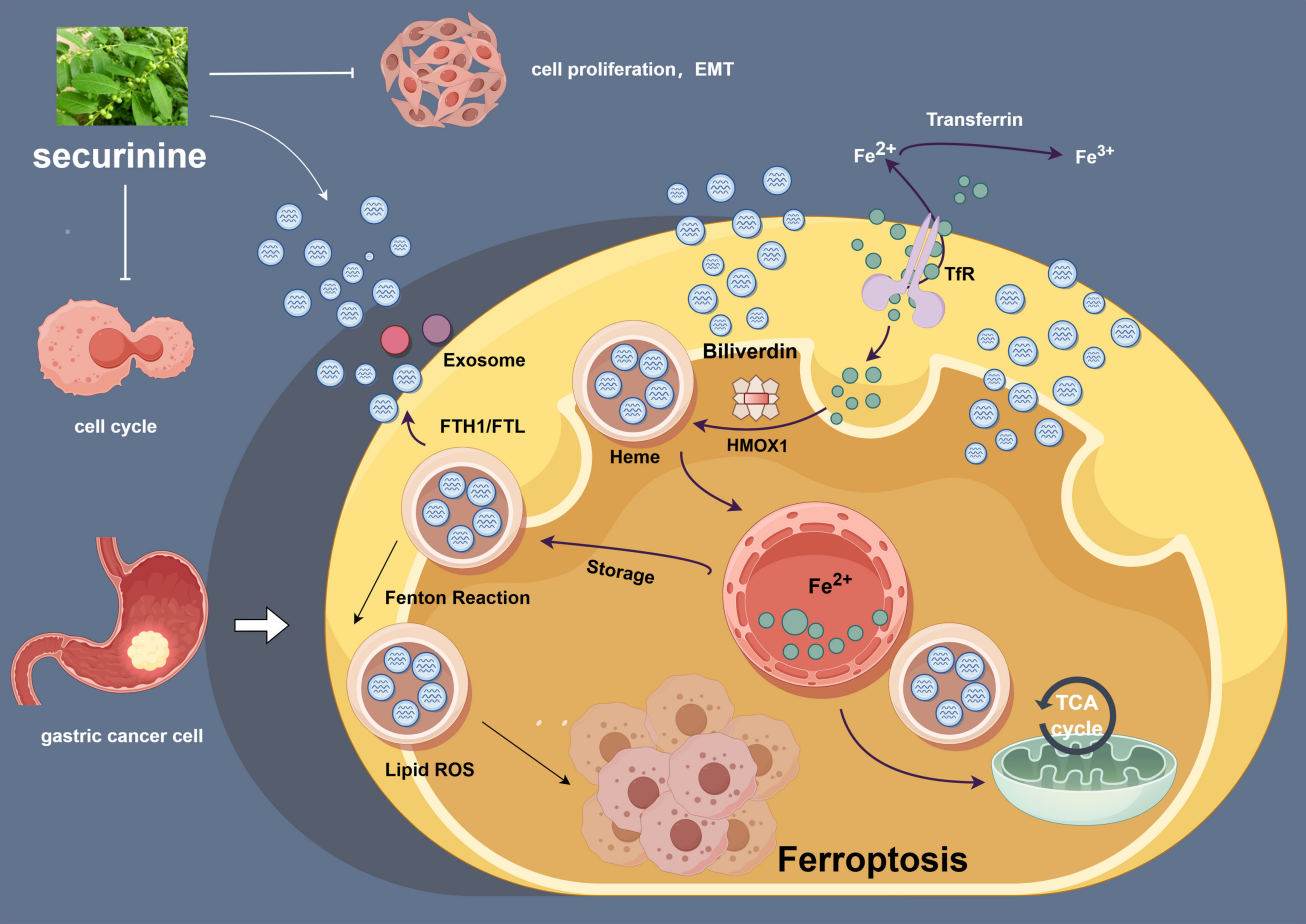
Securinine induces iron-dependent cell death by modulating the expression of HMOX1, FTH1, and FTR, and regulates the cell cycle and epithelial-to-mesenchymal transition (EMT) process in gastric cancer cells. This schematic diagram outlines the mechanism of action of securinine in gastric cancer cells. securinine promotes the accumulation of iron ions by upregulating the expression of HMOX1, FTH1, and FTR, thereby activating iron-dependent cell death (ferroptosis). Additionally, securinine regulates cell cycle-related proteins (such as CDK2, Cyclin D1, and P21) and EMT-related proteins (such as E-CAD, N-CAD, and VIM), inhibiting cell proliferation and inducing the EMT process. This figure reveals the potential role of securinine in inhibiting tumor growth in gastric cancer cells through multiple mechanisms.

In order to further illustrate the proposed mechanisms, we constructed a schematic diagram (**Figure 11**) summarizing the role of securinine in gastric cancer cells.

This figure highlights how securinine promotes iron-dependent cell death by upregulating the expression of key ferroptosis-related genes such as HMOX1, FTH1, and FTR. It also demonstrates securinine's ability to regulate cell cycle proteins (e.g., CDK2, Cyclin D1, and P21) and epithelial-to-mesenchymal transition (EMT) markers (e.g., E-CAD, N-CAD, and VIM), thereby inhibiting cell proliferation and modulating the EMT process. These findings suggest that securinine may inhibit gastric cancer progression through multiple synergistic mechanisms.

## Discussion

Securinega alkaloids, derived from the leaves and roots of the Asian plant Securinega suffruticosa, have attracted widespread attention since their discovery due to their significant biological activities. These indolopyridine alkaloids, particularly their derivatives, have shown considerable potential in the treatment of cancer and neurological diseases (25–27). While the mechanisms of these compounds are not fully understood, existing research has revealed their broad biological effects, including inducing cell differentiation, reversing multidrug resistance, cardiovascular protection, anti-inflammatory actions, serving as vaccine adjuvants, and combating various pathogens (28–30).

Of particular note is securinine, a primary Securinega alkaloid, whose chemically modified derivatives at the C12, C14, and C15 positions have shown enhanced antitumor effects. Studies have found that bivalent analogs linked to the C15 position are particularly effective in inducing cell differentiation and reversing P-glycoprotein-mediated drug resistance (31). Moreover, the modulation of key signaling pathways such as JAK/STAT, PI3K/AKT/mTOR, and MAPK by securinine and its derivatives underscores their critical role in regulating cell growth, differentiation, and death (27). Research into securinine has not only highlighted its potential as a potent excitant in the central nervous system—applicable in the treatment of conditions like amyotrophic lateral sclerosis, multiple sclerosis, and Parkinson’s disease—but also its extensive potential in cancer research. securinine promotes apoptosis through various mechanisms, including mitochondrial dysfunction, reactive oxygen species (ROS) production, and activation of mitogen-activated protein kinases (MAPKs), thereby exerting inhibitory effects on various cancer cells (32). Additionally, the regulation of the PI3K/AKT/mTOR signaling pathway, reduction in the expression levels of Bcl-2, mTOR, and P70S6k, as well as the overexpression of pro-apoptotic proteins like Bax, demonstrate the multi-targeted mechanism of securinine in cancer therapy (33, 34).

In this study, we conducted an in-depth analysis of the capacity of securinine to induce iron-dependent cell death (ferroptosis) in gastric cancer cells. Through experiments in the HGC27 and MGC803 gastric cancer cell lines, we first determined the half-maximal inhibitory concentration (IC50) of securinine and its effective duration, confirming its significant inhibitory effect on gastric cancer cells. Further evaluations using CCK-8 and EDU proliferation assays, as well as Western blot analysis of cell−cycle−related proteins, revealed that securinine effectively blocks the progression of the cell cycle in gastric cancer cells, particularly the G2/M transition. Investigating its mechanism of action, we utilized Western Blot and immunofluorescence techniques to study the impact of securinine on epithelial-mesenchymal transition (EMT). Results indicated that securinine not only inhibits the proliferation of gastric cancer cells but also suppresses their dissemination and invasive capabilities by modulating EMT-related markers. Additionally, subcutaneous tumor formation experiments in nude mice further confirmed the inhibitory effect of securinine on the growth of gastric cancer cells *in vivo*. To delve deeper into the molecular mechanisms of securinine, we conducted whole-genome sequencing analysis of gastric cancer cells, focusing on iron metabolism pathways related to ferroptosis. Through a series of biochemical experiments, including MDA measurements, GSSH assays, mitochondrial ROS determinations, lipid peroxidation probe experiments, and mitochondrial electron microscopy observations, we detailed how securinine promotes iron-dependent cell death. Our experimental results demonstrated that treatment with securinine significantly increased the intracellular iron concentration, ROS production, and lipid peroxidation levels in gastric cancer cells. The induced cell death was not reversed by pre-treatment with apoptosis inhibitor Z-VAD-FMK, necrosis inhibitor Necrosulfonamide, or autophagy inhibitor 3-methyladenine (3-MA), suggesting that the cell death induced by securinine did not involve apoptosis, necrosis, or autophagy. These changes were effectively reversed by ferroptosis inhibitors ferrostatin-1 and Liproxstatin-1. Further, intervention experiments targeting the key iron metabolism gene HMOX1, including gene knockdown and the use of the HMOX1 inhibitor Zinc Protoporphyrin (ZNPP), observed a restoration in the expression of cell cycle and EMT-related proteins, further strengthening the hypothesis that securinine inhibits gastric cancer cell proliferation and EMT processes by activating ferroptosis mechanisms.

In this research, we explored the efficacy of securinine in inducing iron-dependent cell death in gastric cancer cells. Through comprehensive use of *in vitro* and *in vivo* models, we revealed the significant role of securinine in inhibiting the proliferation of gastric cancer cells. More importantly, we discovered that securinine could induce iron-dependent cell death through multiple biological mechanisms, including interfering with the normal operation of the cell cycle, suppressing the process of epithelial-mesenchymal transition (EMT), and regulating iron metabolism. These series of findings not only deepen our understanding of securinine’s anticancer mechanisms but also pave new pathways and targets for gastric cancer treatment research. By observing the characteristics of slowed growth and increased necrosis rates in gastric cancer cells treated with securinine, these changes indicate the potential of securinine in inhibiting tumor cell growth. Further studies on the molecular mechanisms showed that securinine significantly adjusts the expression of key proteins related to the cell cycle, blocking the normal progression of the cell cycle, thereby inhibiting cell proliferation. Additionally, by affecting the expression of key markers in the EMT process, securinine effectively maintained the epithelial characteristics of the cells, slowing down the invasiveness and migration capabilities of the tumor cells. Moreover, securinine’s regulation of iron metabolism, especially by promoting iron accumulation, further triggered iron-dependent cell death, providing an important biological basis for its anticancer activity.

A significant innovation in this study is the elucidation of the mechanism by which securinine induces ferroptosis by regulating the iron metabolism pathway and its application in anti-cancer research for gastric cancer cells. This discovery provides a new perspective on understanding the role of securinine in gastric cancer treatment. Through meticulous experimental design, we revealed how securinine affects the metabolic processes of iron within cells, thereby triggering iron-dependent cell death, a first in the field of gastric cancer cell treatment research. Additionally, this study, by specifically intervening in the expression of the HMOX1 gene, further verified the specific mechanism of securinine-induced ferroptosis, thereby deepening our understanding of the potential and applicability of ferroptosis in inhibiting gastric cancer cell growth and promoting cell death.

Although this study reveals the potential of securinine-induced ferroptosis in the treatment of gastric cancer to some extent, there are still several limitations and areas for improvement. First, although we have used various experimental means to verify that securinine can effectively induce ferroptosis and promote apoptosis in gastric cancer cells, the detailed molecular mechanisms behind this, especially the upstream signaling pathways and core regulatory factors affecting this process, still need to be further explored and clarified. This is important for deepening our understanding of the mechanisms of action of securinine and for future targeted drug development. Second, although this study focused on the inhibitory effects of securinine in gastric cancer cells, the potential efficacy and mechanisms of action of this compound in other types of cancer have not been extensively studied. Different types of cancer, due to their unique biological characteristics and microenvironments, may respond differently to securinine, therefore, expanding future research to more cancer types will help to comprehensively assess the anticancer potential of securinine. Additionally, although *in vivo* experimental data support the effectiveness of securinine in inhibiting the proliferation of gastric cancer cells, systematic research on the safety evaluation of this drug, the most suitable administration scheme, and potential side effects is still lacking. Research in these areas is indispensable before advancing securinine to clinical application. This includes but is not limited to large-scale preclinical safety assessments, pharmacokinetic studies, and optimization of administration strategies, to ensure that securinine achieves the best balance of efficacy and safety in practical applications.

In summary, this study, by exploring the mechanism by which securinine induces iron-dependent cell death in gastric cancer cells, provides scientific evidence for its potential as an anticancer drug. Future research needs to build on this foundation to further explore its potential in clinical treatment, including optimizing efficacy, comprehensively analyzing the mechanisms of action, and assessing safety.

## Conclusions

This study successfully reveals how securinine exerts significant inhibitory effects in gastric cancer cells by regulating iron metabolism pathways to activate iron-dependent cell death. These findings not only highlight the substantial potential of securinine in the field of cancer therapy but also emphasize the importance of understanding and utilizing the mechanism of ferroptosis in developing treatment strategies for gastric cancer. Additionally, the results further confirm the pivotal role of iron metabolism in the progression and therapeutic targeting of tumors, paving the way for novel strategies that target iron metabolism for gastric cancer treatment. Through detailed analysis of securinine’s modulation of iron metabolic pathways, this study enhances our understanding of the complex mechanisms of iron-dependent cell death.

Furthermore, these findings indicate new directions for the treatment of gastric cancer and potentially other cancers, showcasing the key role that securinine and its derivatives could play in future cancer therapy strategies. By exploring the regulatory mechanisms of iron metabolic pathways and their role in the process of tumor cell death, this research provides strong theoretical support and experimental evidence for the development of new anticancer therapies based on iron metabolism. This not only promises more effective treatment options for patients with gastric cancer but may also impact the treatment of other cancers. In summary, the discoveries of this study are not only scientifically significant but also have profound implications for the clinical application of future cancer treatments, underscoring the importance of securinine in cancer research and therapeutic practices.

## Data Availability

The data in this study are available from the corresponding author upon reasonable request. Requests to access these datasets should be directed to Weiwei Yuan at yww15391960716@163.com.

## Glossary

ALP: alkaline phosphatase
ANOVA: analysis of variance
CCK-8: Cell Counting Kit-8
CDK2: Cyclin-dependent kinase 2
DAPI: 4′,6-diamidino-2-phenylindole
EdU: 5-ethynyl-2′-deoxyuridine
EMT: epithelial-mesenchymal transition
Fer-1: ferrostatin-1
FTH1: ferritin heavy chain 1
FTR: ferritin light chain
GAPDH: glyceraldehyde-3-phosphate dehydrogenase
G1/S: G1 to S phase
G2/M: G2 to M phase
GSH: glutathione
GPX4: glutathione peroxidases 4
GSEA: gene set enrichment analysis
HE: hematoxylin and eosin
HMOX1: heme oxygenase 1
IF: immunofluorescence
JAK: Janus kinase
KEGG: Kyoto Encyclopedia of Genes and Genomes
Liperfluo: lipid peroxide probe
MDA: malondialdehyde
MGC803: a gastric cancer cell line
PCA: Principal Component Analysis
ROS: reactive oxygen species
siGPX4: siRNA against GPX4
siNrf2: siRNA against Nrf2
siSLC7A11: siRNA against SLC7A11
scr: scramble RNA
SLC7A11: solute carrier family 7 member 11
SPR: surface plasmon resonance
TEM: transmission electron microscopy
WB: Western Blot
ZNPP: Zinc Protoporphyrin
