# Supplementary material for: Securinine, a novel alkaloid, regulates cell cycle and EMT in gastric cancer by inducing iron-dependent cell death
Source: Front Oncol. 2025 Sep 10;15:1599680. doi: 10.3389/fonc.2025.1599680 (PMC12457131; doi:10.3389/fonc.2025.1599680)
Supplement: Supplementary file 1 [file Table1.docx]

**siRNA sequences**

| **primer names** | **sequence (5' to 3')** |
| --- | --- |
| HMOX1(human）siRNA-671 | GGGUGAUAGAAGAGGCCAATT |
|  | UUGGCCUCUUCUAUCACCCTT |
| HMOX1(human）siRNA-800 | GCAACAAAGUGCAAGAUUCTT |
|  | GAAUCUUGCACUUUGUUGCTT |
| HMOX1(human）siRNA-161 | CAGAGAAUGCUGAGUUCAUTT |
|  | AUGAACUCAGCAUUCUCUGTT |
| negative control | UUCUCCGAACGUGUCACGUTT |
|  | ACGUGACACGUUCGGAGAATT |
| FAM negative control | UUCUCCGAACGUGUCACGUTT |
|  | ACGUGACACGUUCGGAGAATT |
| positive control (human GAPDH) | GUAUGACAACAGCCUCAAGTT |
|  | CUUGAGGCUGUUGUCAUACTT |
